# 

**Published:** 1911-02-25

**Authors:** 


### CONTENTS.

| | PAGE |
|---|---|
| **The Tropics and Life Assurance** | 623 |
| **Variation and Environment in Disease** | 624 |
| **Annotations**—A Notice—The Future of the White Races—The Meat and Milk Supply—Truth's Cautionary List | 625 |
| **Medical Opinion and Movement** | 626 |
| **Hospital Clinics**—Gas in the Stomach; Its Causation and Treatment. By Frank Kennedy Cahill, F.R.C.S., D.P.H. | 629 |
| **Medicine**—Jaundice in Secondary Syphilis—Hæmaturia and Nephritis of Sudden Onset as an Initial Symptom of Typhoid Fever | 631 |
| **Dermatology**—Cutaneous Diphtheria | 632 |
| **Surgery**—The Surgical Uses of Iodine | 633 |
| **Tropical Medicine**—A Probable New Human Trypanosome | 634 |
| **Special Articles**—Home and Continental Spas | 635 |
| **Parliament and Publications** | 637 |
| **Editor's Letter Box** | 638 |
| **Answers to Correspondents** | 638 |
| **News and Events of the Week** | 639 |
| **Medical Appointments, &c.** | 640 |
| **Obituary** | 640 |
| **The Week in the Courts** | 640 |
| **The Week's Work** | 641 |
| **The Hospital World** | 642 |
| **Gifts and Bequests** | 642 |
| **Jottings** | 642 |
| **Hospital Architecture and Construction** | 643 |
| **A New Type of Motor Ambulance** | 644 |
| **Institutional Notes and News** | 645 |

